# Medical and Physical Intelligence

**Published:** 1805-04-01

**Authors:** 


### MEDICAL AND PHYSICAL INTELLIGENCE.

### [ FOREIGN AND DOMESTIC. ]

### NON-CONTAGIOUSNESS OF YELLOW FEVER.

*Neither the Black-vomit, Serum, nor Saliva of Persons labouring under the Yellow Fever, capable of communicating that Disease.* — Dr. Ffirth, of Salem, in New-Jersey, has published a dissertation on malignant fever, with an attempt to prove that it is not contagious. In this he relates a number of experiments which he has made upon the matter of black-vomit, as discharged by persons labouring under that disease. These experiments are bold and decisive; and their author deserves the thanks of the civilized world for having made them. They prove that the dark-coloured fluid voided from the stomachs of those who are suffering the latter symptoms of that malady, is wholly innoxious. They agree in the main with the experiments on the same matter which were related in the Med. Repository of New York, vol. iv. p. 163. But this intrepid gentleman has carried them further than those, and, indeed, far beyond the trials of any other person. We recommend them to the careful consideration of the contagionist here and elsewhere.

Dr. Ffirth fed a dog for a week on bread soaked in black-vomit, and on the ejected black-vomit matter itself. He grew fond of his food, and enjoyed very good health. A cat was kept on similar diet for five days. She lapped the black matter with avidity, and had no symptom of disease. Fresh black-vomit was inserted between the skin and muscles of a dog's back, and confined there. The wound healed by the first intention, the fluid was absorbed, and the creature continued healthy after this extraordinary inoculation. Dr. Ffirth then inoculated himself in the left fore-arm with black-vomit just discharged from a moribund patient; a slight inflammation ensued, which subsided in three days, and the wound readily healed. He confined some of the same dark matter, immediately after ejection, over a cut in his right arm, by sticking-plaster, for two days. On examination at the end of that time, there was no more inflammation discoverable than if the black-vomit had not been introduced. The wound readily healed, and without the formation of pus. To avoid cavil and deception, he repeated these experiments above twenty times, on various parts of his body, with the black matter collected in Philadelphia during the seasons of 1802 and 1803. He put it into his eye without experiencing more inconvenience than cold water produces. He exposed himself to the exhalations of it, while acted upon by heat in an iron skillet, and and experienced no unpleasant sensation. He swallowed the thick extractive matter, which remained after evaporation, in the form of pills, without incommoding his stomach. He even went so far as to mix half an ounce of fresh black-vomit with an ounce and a half of water and to drink it. It produced no more effect on his stomach than so much water. He increased the dose to two ounces; and finally swallowed the black-vomit in like quantity, without any dilution at all, and without sustaining the least injury. He inoculated himself with saliva and serum with as little inconvenience!

---

The following Annual Report of a National Institution in Ireland for the Extermination of the Small-Pox, was transmitted to the Editors by Dr. WALKER, of Salisbury Square.

"On the Business of the Royal Jennerian Society,
"The Secretary,
"14, Salisbury Square.

"Sir,

"I am directed to transmit to you the enclosed Report of our Institution, the copy of an advertisement, &c. and to request that you may forward to us whatever papers you may have published upon the subject of cow-pock.

"I am, &c.

*Feb.* 26, 1805. "S. B. LABATT.

### COW-POCK INSTITUTION,

NORTH COPE STREET, DUBLIN,

*Under the Patronage of His Excellency Earl Hardwicke, &c. &c.*

Opened on January 14, 1804, under the superintendance of the undersigned physicians and surgeons of this city, for the purposes of securing a succession of cow-pock matter, of inoculating gratuitously the children of the poor; and of supplying the different parts of the kingdom with genuine infection.

| *Physicians.* | *Surgeons.* |
| --- | --- |
| JOSEPH CLARKE, | GEORGE STEWART, |
| JAMES CLEGHORN, | RALPH S. OBRE, |
| THOMAS EVORY. | SOLOMON RICHARDS. |

*An Abstract from the Register of Inoculations and Distribution of Matter:*

| | |
| --- | --- |
| Patients inoculated during the last year - - - - | 578 |
| Packets of infection issued to practitioners in general | 776 |
| Packets of infection issued to army surgeons - - | 236 |

In the above five hundred and seventy-eight cases the cow-pock observed its characteristic mildness, not proving fatal in a single instance, nor even inducing a severe disease.

In

In two cases, convulsions appeared on the eighth day, but they were evidently produced by costiveness, on the removal of which the fits subsided.

In a few patients the inoculated part (from being irritated) ran on to ulceration, which however was soon relieved.—An ointment composed of one part of ung. hyd. nit. and two of ung. simp. was found most useful in such cases.

No instance occurred of eruptions over the body.—In two cases the children, by scratching the inoculated parts, conveyed the virus to the under eye-lid, where an adventitious vesicle was thus excited, producing much pain and intolerance of light, which however soon disappeared.

Constitutional symptoms were seldom observed; when such did occur, they were but slight and transient.

Delicate children, and those affected with cutaneous eruptions, (particularly with itch) were found more difficult to be infected with cow-pock than others.

No consequent disease has been observed imputable to cow-pock.

No case occurred to excite the smallest doubt as to the *permanent* efficacy of cow-pock, in protecting the system against the small-pox; neither in this institution, nor in the private practice of any of the gentlemen who superintend it.

Strict attention has been paid to investigate several cases of reputed failure of cow-pock, in preventing small-pox, in this city and its neighbourhood, all of which proved, on minute enquiry, to be the effects of ignorance or misrepresentation.

The increasing applications for cow-pock matter, from the country parts of this kingdom, evince that the new inoculation is gaining ground rapidly, and sanguine hopes are entertained that it will soon become as generally practised in Ireland as in Great Britain.

Signed by order,

S. B. Labatt, M. D. Secretary.

*Dublin, Jan.* 15, 1805.

---

Mr. Goldson's pamphlet having tended to raise fresh doubts in the minds of the already prejudiced against vaccination in this country, it was thought expedient to send several copies of this paper to every county town in Ireland.

*Cow-Pock Institution, North Cope Street, Dublin.*

An opinion having been lately circulated, that cow-pock inoculation ceases, at the expiration of three years, to secure the constitution against the action of small-pox, we think it our duty to inform the public, that all the trials we have hitherto made conspire to prove this opinion erroneous.—In the cases which gave rise to it, we believe there must have been inaccuracy in the choice of the infection employed.

By the most moderate computation, it appears that the number of deaths from small-pox in Great Britain and Ireland still amounts amounts to between *thirty and forty thousand* annually; a practice which bids fair to put a stop to the ravages of so destructive a disease, we think well entitled to a patient and unprejudiced investigation:—Having no object in view but truth and utility, the public may rest assured, should any thing really adverse to cow-pock inoculation occur to us, we shall be among the first to give notice of it.

JOSEPH CLARKE, GEORGE STEWART,

JAMES CLEGHORN, RALPH OBRE,

THOMAS EVORY, SOLOMON RICHARDS.

Signed by Order,

*June* 26, 1805. S. B. LABATT, Secretary.

---

HOWEVER trifling it may appear in the eye of philosophy to mark the coincidence, yet a Correspondent seems anxious to have it noticed, that the 19th of January, 1803, which gave birth to the Royal Jennerian Society in London, was the day on which the Right Hon. the Governor in Council at Madras, was pleased in an Advertisement to announce to the native inhabitants the important advantages to be derived from vaccination, and the encouragement which his Lordship in Council proposed to afford for the extension of so signal a blessing throughout the territories under the Presidency of Fort St. George.

It is our pride to have contributed to the formation of that society, whose services to the world are so honourably recognised by the governors there, as well as by the government at home; more particularly, the War Office and His Majesty's Post Masters General, continue to afford to it a patronage truly liberal.

The Reports we have published from India make the numbers amount to more than 150,000 already protected by the discovery of Jenner.

In the following extracts from some late communications from Dr. Anderson, (through the medium of Dr. Walker) we are happy to find that his zeal remains undiminished, we also obtain from them information both interesting and curious under different points of view; among other pleasing circumstances, let our constant correspondent the indefatigable Mr. Ring, derive an unmixed gratification from learning that his writings, together with those of the Society in which he incessantly labours, receive the *imprimantur* of the Physician General at Madras.

" *To the Editor of the Government Gazette.*

" Sir,

" As the inoculation of cow-pock has surmounted all opposition over the whole of Europe, and the attempts to introduce it here have been attended with reasonable success; there can be but little doubt, that a steady attention to the practice may be productive of the extirpation of small-pox, and such persons as are restrained by the fear of offending Mare Ummœ, the goddess of small-pox in this country, will in due time acquiesce in the use of cow-pock inoculation, inoculation, as well as those who entertained the notion of diseases being a dispensation of Divine Providence in that; all of whom will now thankfully acknowledge a beneficent Deity, permitting human capacity to discover and estimate laws of nature, or second causes, for the welfare of society.

"You will therefore be so good as insert this letter in your Weekly Paper, together with the accompanying table, extracted from p. 1036 of Mr. Ring's History of Vaccine Inoculation, as a compendious calculation of the advantages that will attend the extirpation of small-pox; besides the pleasing gratification of preserving the human race from a dire disease, it may be worthy of remark, that every life here, as a source of revenue to government, may be valued at eight shillings sterling a year; so that, saving the lives of the inhabitants of this country, appears no less an object well meriting every effort, whether it is considered in a philanthropic or pecuniary point of view.

"As it is possible on the extended scale which it is to be hoped vaccination may now come into practice, that many operations may be performed without a proper effect; I have subjoined the signs of infection and description of the vaccine vesicle, as laid down by the Jennerian Society, whereby your readers may be enabled to judge by inspection and examination on the days specified, whether the disease is genuine or not, excepting that in some of the darker complexioned Asiatics, the areola is not so obvious to the eye, as in European patients, on account of the different opacity of the rete mucosum of the skin; but the hand applied to the circumference of the vesicle, readily discovers the firm hardness of the areola in them as well as in Europeans.

"I am, Sir,

"Your obedient and very humble Servant,

"James Anderson, Physician Gen."

*Fort St. George, Aug.* 29, 1804.

"*To the Editor of the Government Gazette.*

"Sir,

"The intellectual powers of the Indians being guided by ancient tradition, appear to advantage in their adoption of cow-pock inoculation; and every remarkable instance of it, as tending to promote the extermination of small-pox, is worthy of notice.

"The Raja of Chintapilly nobly submitted to small-pox inoculation, and His Excellency the Raja of Tanjore has already displayed distinguished countenance in favor of cow-pock inoculation; but it seems to have been reserved for the Dewan of Travancore to submit his own person to so great a novelty.

"Progress towards the extermination of small-pox is become so promising, that I have little doubt, the cow-pock lancet will soon be as familiar to the Hindoos, as the plough or the shuttle.

"The following extract of a letter from Mr. Henry Robertson, Surgeon

Surgeon at Angengo, under date the 3d instant, will therefore prove acceptable intelligence to your readers.

"I beg to assure you, that I shall continue to promote Vac-
" cination by every means in my power—those who have hitherto
" submitted to it in this part of the country, have been chiefly
" Christians and Hindoos of low cast; but the period is fast ap-
" proaching when I trust every inhabitant of Travancore, from
" the highest to the lowest, will lay aside all prejudice, and by
" becoming proselytes to the Jennerian system, will cheerfully
" suffer themselves to be inoculated—there is every reason to
" hope, that this desirable object may be attained, so far at least
" as the powerful influence of example will tend to that effect,
" as the Dawan was vaccinated by Mr. Macauley, at Allepi,
" upon the 30th ultimo."

I am, &c.

JAMES ANDERSON, Physician General.

*Fort St. George, Sept.* 12, 1804.

---

Six of the most eminent physicians in France, Chaussier, Leclerc, Baily, Husson, Nysten, and Hamel, have been sent to Spain, to inquire into the nature of the epidemical disease, which has raged with such violence in that country, and to endeavour to find out the most effectual remedy for preventives against it. The king of Prussia has likewise sent thither, for the same purpose, Prof. Reich, of Erlangen.

---

The Icelandic moss, which has lately been discovered in Spain, has likewise been found in the district of Concossola, in the Italian republic.

---

The society for the encouragement of arts have lately voted their gold medal to Dr. Howison for his preparation of tan made in the East Indies from the bark of the mangrove tree.

---

Mr. Young, surgeon, of North Audley Street, will publish early in May, an important work on the subject of Cancer, entitled *Sanaschirrologia;* containing an analytical inquiry into the nature and action of Schirrus, in order to establish a regular mode of curing that disease in its various stages, by means of natural separation.

---

Mr. ROGERS has just published a work, entitled, "An Examination of that Part of the Evidence relative to Cow-pox, which was delivered to a Committee of the House of Commons by two of the Surgeons of St. Thomas's Hospital: With Remarks on the inoculated Small-pox."

---

The Papers of Mr. Clement, Mr. Dunning, and Chirurgicus, intended to have been inserted in this month's Journal, are unavoidably deferred till our next.

